# Distribution and determinants of patient satisfaction in oncology with a focus on health related quality of life

**DOI:** 10.1186/1472-6963-9-190

**Published:** 2009-10-21

**Authors:** Christopher G Lis, Mark Rodeghier, James F Grutsch, Digant Gupta

**Affiliations:** 1 Cancer Treatment Centers of America^® ^(CTCA) at Midwestern Regional Medical Center, 2610 Sheridan Road, Zion, IL, 60099, USA

## Abstract

**Background:**

Cancer patients usually undergo extensive and debilitating treatments, which make quality of life (QoL) and patient satisfaction important health care assessment measures. However, very few studies have evaluated the relationship between QoL and patient satisfaction in oncology. We investigated the clinical, demographic and QoL factors associated with patient satisfaction in a large heterogeneous sample of cancer patients.

**Methods:**

A cohort of 538 cancer patients treated at Cancer Treatment Centers of America^® ^(CTCA) was assessed. A patient satisfaction questionnaire developed in-house by CTCA was used. It covered the following dimensions of patient satisfaction: hospital operations and services, physicians and staff, and patient endorsements for themselves and others. QoL was assessed using the European Organization for the Research and Treatment of Cancer Quality of Life Questionnaire (QLQ-C30). The clinical, demographic and QoL factors were evaluated for predictive significance using univariate and multivariate logistic regression.

**Results:**

The mean age of our patient population was 54.1 years (SD = 10.5, range 17-86), with a slight preponderance of females (57.2%). Breast cancer (n = 124) and lung cancer (n = 101) were the most frequent cancer types. 481 (89.4%) patients were "very satisfied" with their overall experience. Age and several QoL function and symptom scales were predictive of overall patient satisfaction upon univariate analysis. In the multivariate modeling, only those with a score above the median on the fatigue measure (i.e. worse fatigue) had reduced odds of 0.28 of being very satisfied (p = 0.03).

**Conclusion:**

Patient fatigue, as reported by the QoL fatigue scale, was an independent significant predictor of overall patient satisfaction. This finding argues for special attention and programs for cancer patients who report higher levels of fatigue given that fatigue is the most frequently reported symptom in cancer patients.

## Background

In recent years, awareness has risen of how patients perceive the quality of their care [1,2]. In an extremely competitive environment, patient satisfaction has become a tool to gain attention and value amongst the patients as well as providers. Hospitals and other health care centers are increasingly using this information while making important decisions regarding the operational and treatment plans [3]. The patient satisfaction data is the key indicator of the quality of care and treatment delivered by the physicians, paramedical staff and the hospital as a whole [4]. The health centers can use survey results to design and track quality improvement over time, as well as compare themselves to other health centers. Also, this information is of great use for healthcare accreditations. By conducting their own surveys, the health care organizations are able to recognize and resolve potential patient satisfaction problems and thus improve their strategies [5,6]. Having satisfaction surveys also helps identify the specific needs of the patients for the health care provider [7].

The assessment of patient satisfaction in an oncology setting is particularly salient. Advances in diagnostics, treatment, supportive care and rehabilitation all necessitate continued monitoring to determine whether patients are satisfied with the increasingly complex and multidisciplinary nature of health care services that they are receiving, and to identify areas in which improvement is needed. Cancer patients should be surveyed regularly due to the usual extensive and debilitating treatments that they must undergo. The modes of therapies have their own side effects and often result in difficult patient compliance. As a result, considerable demands are placed on health care providers to satisfy the complex healthcare needs of cancer patients. Several studies have been done to study patient satisfaction in cancers like gastro esophageal [8], breast [5,9], colorectal [10], lung, prostate [11] and gynecological [12,13]. But very few have dealt with a large sample size and a population that is heterogeneous with respect to cancer types.

Similarly, a number of studies have been conducted to evaluate the predictors of patient satisfaction in various healthcare settings including oncology [14-18]. Patient perceptions of needs met or emotional support provided were predicted by their perceptions of the occurrence of physician behaviors involving information such as the diagnosis and tests and treatment [19]. Patient perceptions of physician behaviors were found to be stronger predictors of patient satisfaction than the actual occurrence or absence of those behaviors [20]. A poorer prognosis and a positive quality of the day's news were associated with higher satisfaction. Use of the patient's first name and attempts to establish privacy during an exam were positively correlated with satisfaction, whereas discussing the role of the family and tumor status had a negative impact [21,22]. The number of nurses and doctors per bed, institution size, geo-cultural origin, ward setting, teaching/non-teaching setting, treatment toxicity, global health status, participation in clinical trials and education level were all associated significantly with satisfaction. Doctor's and nurse's interpersonal skills, information provision, and availability also influenced overall satisfaction [23,24]. Common predictors of the overall quality perception which the patients perceive as relatively problematic aspects of care were 'was informed about follow-up care after completing treatment', 'knew next step in care', 'knew who to go to with questions', and 'providers were aware of test results'. The type of treatment received and duration of disease also influenced the level of patient satisfaction [25,26]. Moreover, patient satisfaction can at times be affected by the resistance to the lifestyle changes that a cancer diagnosis and treatment entails and might not necessarily be a reflection of the patients' perceptions of care with their healthcare providers. There also have been a few studies that have indicated a possible link between quality of life (QoL) and patient satisfaction in cancer [27-31].

There is extensive data in the literature demonstrating the use of QoL tools as important predictors of patient outcomes such as survival [32-37]. QoL tools measuring the activities of daily life can predict survival in several different types of cancers independent of the extent of the disease and other clinical prognostic factors. These studies have used different combinations of clinical and QoL factors in multivariate models evaluating the prognostic significance of each on clinical outcomes. These studies have used a variety of QoL tools to measure the activities of daily living, the most commonly used instrument being the European Organization for Research and Treatment of Cancer Quality of Life Questionnaire (QLQ-C30).

In the light of above observations, we hypothesized that QoL tools could predict patient satisfaction with the care received in an oncology treatment setting. The goal of this study was two fold: to describe the patients' experiences with the care they receive and to investigate the clinical, demographic and QoL factors associated with patient satisfaction in a large heterogeneous sample of cancer patients treated at a community hospital comprehensive cancer center. For the purpose of this study, the definition of QoL was based on criteria in the QLQ-C30 questionnaire, which focuses on the patient's capacity to fulfill the activities of daily living.

## Methods

### Study Population

A cohort of 538 cancer patients treated at Cancer Treatment Centers of America^® ^(CTCA) at Midwestern Regional Medical Center (MRMC) and Cancer Treatment Centers of America^® ^at Southwestern Regional Medical Center (SRMC) between August 2006 and December 2007 was surveyed. All patients who had completed a treatment plan consultation with a CTCA physician without having undergone any treatment at CTCA were eligible to participate in this study. Patients with all stages of all cancer types were eligible for the study. The study was approved by the Institutional Review Boards at MRMC and SRMC.

### Questionnaire

The questionnaire was first developed and implemented by the Research team at CTCA in August 2006. The survey was administered daily at both CTCA sites. An attempt was made to approach all new patients onsite for survey administration. Those who were not approached onsite were sent a survey in the mail. The survey, return envelope and a cover letter was provided in these mailings to the patients.

In brief, our patient satisfaction questionnaire relates to the following dimensions of patient satisfaction: hospital operations and services, physicians and staff, and patient endorsements for themselves and others. The majority of the questions were measured on a five-point scale from "Very dissatisfied" to "Very satisfied". The patients were asked about their method of contact with our hospital as well as the primary reason of their visit. Finally, the patients were asked if our hospital met their expectations and if they would bring their relatives and friends to the hospital if needed.

### QoL Assessment

QoL was assessed using QLQ-C30, which emphasizes a patient's capacity to fulfill the activities of daily living. The QLQ-C30 is a 30-item cancer specific questionnaire that incorporates five functioning scales (physical, role, cognition, emotional, and social), nine symptom scales (fatigue, pain, and nausea/vomiting, dyspnea, insomnia, loss of appetite, constipation, diarrhea, financial problems), and a global health status/QoL scale. The raw scores are linearly transformed to give standard scores in the range of 0-100 for each of the functioning and symptom scales. Higher scores in the global and functioning scales and lower scores in the symptom scales indicate better QoL. A difference of 5-10 points in the scores represents a small change, 10-20 points a moderate change and greater than 20 points a large clinically significant change from the patient's perspective [38]. This instrument has been extensively tested for reliability and validity [39-41] and is one of the most common QoL questionnaires in cancer research.

### Statistical Analysis

All data were analyzed using SPSS version 16.0 (SPSS, Chicago, IL, USA). Descriptive statistics and frequencies were computed for each item in the questionnaire. Stage at diagnosis was categorized into 2 groups of stages I and II (early stage) and stages III and IV (late stage). The prior treatment history variable categorized the patients into those who have received definitive cancer treatment elsewhere before coming to our institution and those who were newly diagnosed at our institution. Study patients were dichotomized into two groups based on the median scores for all QoL scales to yield "above median" and "below median" scores. This was done to reduce multicollinearity between various QoL scales. The median score was chosen arbitrarily as there are no established cutoffs for QoL in cancer patients. A difference was considered to be statistically significant if the p value was less than or equal to 0.05. Distribution of patient satisfaction analyses was based on a sample size of 538 while the predictors of patient satisfaction analyses were based on a sample size of 445 only. This is because QoL data was available for only 445 out of 538 patients.

The available clinical factors and demographic variables, as well as QoL, were evaluated for predictive significance using either t-tests or chi-square tests, as appropriate. A difference was considered to be statistically significant if the p value was less than or equal to 0.05. The question "Considering everything, how would you rate your overall experience with CTCA" was used as the dependent variable. It was measured on a five-point scale from "Very dissatisfied" to "Very satisfied. For the purpose of this analysis, it was dichotomized into 2 groups: "very satisfied" and all other categories combined into "not very satisfied". Multivariate logistic regression analyses were then performed to evaluate the joint prognostic significance of those clinical, demographic and QoL factors that were shown to be predictive in univariate analyses. In particular, QoL scales found to be significant upon univariate analysis were evaluated for their association with patient satisfaction both with and without controlling for the clinical and demographic factors. Each QoL scale was treated as a dichotomous variable for the purpose of univariate and multivariate logistic regression analyses.

## Results

### Response Rate

A total of 2203 patients were contacted at both centers combined to participate in the survey between August 2006 and December 2007 at their initial visit to CTCA. However, only 538 patients responded. As a result, the response rate for this study was 24.4%. 62.6% of surveys were collected in-person; 37.4% via mail, with no difference in overall satisfaction by mode of data collection (p = 0.3).

### Baseline Patient Characteristics

Table 1 shows baseline patient characteristics. The mean age of our patient population was 54.1 years (SD = 10.5, range 17-86), with a slight preponderance of females (57.2%). Breast cancer (n = 124, 23.0%) and lung cancer (n = 101, 18.8%) were the most frequent cancer types found in our study population.

**Table 1:** Patient characteristics (N = 538)

**Variable**	**Categories**	**N**	**Percent**
CTCA Site	Midwestern	342	63.6
	Southwestern	196	36.4

Gender	Male	230	42.8
	Female	308	57.2

Prior Treatment History	Newly Diagnosed	235	43.7
	Previously Treated	303	56.3

Tumor Type	Breast	124	23.0
	Lung	101	18.8
	Colorectal	60	11.2
	Prostate	58	10.8
	Pancreas	46	8.7
	Ovary	19	3.5
	Others	130	24.2

Stage at Diagnosis	I	55	10.2
	II	134	24.9
	III	115	21.4
	IV	174	32.3
	Unknown	60	11.2

### Patient Satisfaction Responses

Table 2 describes the level of patient satisfaction with CTCA operations and services. 481 (89.4%) patients were "very satisfied" with their overall experience with CTCA. A high proportion of patients were "very satisfied" with scheduling of their first visit (n = 475, 88.5%) and the speed of admissions and registrations (n = 483, 89.8%). Relatively smaller number of patients (n = 355, 67%) were "very satisfied" with the amount of time they had to wait for the appointments. Table 3 describes the level of patient satisfaction with CTCA physicians and staff. All physician satisfaction items had a "very satisfied" rating of over 80%. Satisfaction with the staff was high with 92.9% of the patients "very satisfied". Table 4 reports the patient endorsement of CTCA for themselves and others. 468 (87%) patients said they would "definitely" bring their mother, father or other loved ones to CTCA for treatment while 459 (85.3%) patients said they were "extremely likely" to recommend CTCA to friends and associates.

**Table 2:** Patient satisfaction with CTCA operations and services

**Item**	**VD**		**SD**		**N**		**SS**		**VS**	
	
	**N**	**%**	**N**	**%**	**N**	**%**	**N**	**%**	**N**	**%**
Considering everything, how would you rate your overall experience with CTCA?	7	1.3	2	0.4	2	0.4	35	6.5	481	89.4

How satisfied were you with the convenience of your transportation arrangements?	1	0.2	7	1.3	26	4.8	43	8.0	415	77.1

How satisfied were you with the scheduling of your first visit to CTCA?	8	1.5	4	0.7	8	1.5	42	7.8	475	88.5

How satisfied were you with the speed of the registration process?	3	0.6	3	0.6	8	1.5	40	7.4	483	89.8

How satisfied were you with the amount of time you had to wait for appointments?	5	0.9	16	3.0	24	4.5	13	24.5	355	67.0

VD = Very Dissatisfied

SD = Somewhat Dissatisfied

N = Neutral

SS = Somewhat Satisfied

VS = Very Satisfied

**Table 3:** Patient satisfaction with physicians and staff

**Item**	**VD**		**SD**		**N**		**SS**		**VS**	
	**N**	**%**	**N**	**%**	**N**	**%**	**N**	**%**	**N**	**%**

Helping you understand your medical condition	3	0.6	3	0.6	7	1.3	64	11.9	457	84.9

Explaining your treatment options to you	3	0.6	3	0.6	10	1.9	67	12.8	441	84.2

Time that you were able to spend with our doctor(s)	1	0.2	8	1.5	11	2.0	65	12.1	446	82.9

Overall satisfaction with our physicians	3	0.6	4	0.7	5	0.9	46	8.6	470	87.4

Our staff caring for you as an individual	3	0.6	4	0.7	5	0.9	26	4.9	496	92.9

VD = Very Dissatisfied

SD = Somewhat Dissatisfied

N = Neutral

SS = Somewhat Satisfied

VS = Very Satisfied

**Table 4:** Patient endorsement of CTCA for themselves and others

**Item**	**Categories**	**N**	**%**
Will you return for treatment?	No	6	1.1
	Undecided	19	3.5
	Yes	480	85.5

Would you bring your mother, father or other loved ones to CTCA for treatment?	Definitely not	4	0.7
	Probably not	1	0.2
	Not Sure	6	1.1
	Probably	45	8.4
	Definitely	468	87.0

Will you recommend CTCA to friends and associates?	Not at all likely	1	0.2
	1	1	0.2
	2	1	0.2
	4	1	0.2
	Neutral	3	0.6
	7	5	0.9
	8	17	3.2
	9	43	8.0
	Extremely Likely	459	85.3

### Univariate Analysis - Predictors of Patient Satisfaction

Table 5 describes the means, medians and standard deviations of QLQ-C30 scale scores. Among the QLQ-C30 functioning scales, the emotional functioning scale had the lowest (worst) mean score of 67.0 while the highest (best) mean score of 79.3 was recorded for the cognitive functioning scale. Among the QLQ-C30 symptom scales, the nausea scale had the lowest (best) mean score of 10.4 while the highest (worst) mean score of 37.4 was recorded for the fatigue scale.

**Table 5:** Baseline QLQ-C30 scores of 445 cancer patients

**QLQ-C30 Scale**	**Mean**	**Median**	**Standard Deviation**	**Range**
Global	60.8	66.6	25.9	0-100
Physical	78.4	86.6	22.5	0-100
Role	69.6	83.3	33.2	0-100
Emotional	67	66.6	24.3	0-100
Cognitive	79.3	83.3	24.7	0-100
Social	70.8	83.3	31.3	0-100
Fatigue	37.4	33.3	27.8	0-100
Nausea/Vomiting	10.4	0.0	18.7	0-100
Pain	31.7	16.6	31	0-100
Dyspnea	21.4	0.0	28.6	0-100
Insomnia	37	33.3	32.4	0-100
Appetite Loss	33.4	0.0	30.6	0-100
Constipation	17.6	0.0	27.2	0-100
Diarrhea	12.28	0.0	22.6	0-100
Financial Difficulty	26.8	0.0	32.9	0-100

Table 6 describes the univariate analysis of clinical and demographic predictors of patient satisfaction. There were no significant predictors of patient satisfaction as shown in Table 6. The only significant predictor was age at diagnosis, with mean age of 49.3 years for "not very satisfied" category and 54.0 years for "very satisfied" category (p = 0.008).

**Table 6:** Univariate analysis of clinical and demographic predictors of patient satisfaction

**Variable**	**Not very satisfied** **(%)**	**Very satisfied** **(%)**	**P - Value**
Gender			
Male	9.9	90.1	0.2
Female	6.6	93.4	
Prior Treatment History			
Newly diagnosed	5.7	94.3	0.2
Previously treated	9.8	90.2	
Tumor Type			
Breast	8.0	92.0	
Colorectal	4.1	95.9	
Lung	3.7	96.3	
Ovary	0.0	100.0	0.1
Pancreas	17.9	82.1	
Prostate	8.0	92.0	
Other	10.8	89.2	
Stage at Diagnosis			
Early	6.2	93.8	0.4
Late	8.7	91.3	
Age at Diagnosis (Mean)	49.3 years	54.0 years	0.008

Table 7 displays the univariate analysis of QoL predictors of patient satisfaction. The QoL functioning scales that were found to be significant were global, physical, role and social whereas the significant symptom scales were fatigue, nausea, pain, appetite loss and financial difficulty.

**Table 7:** Univariate analysis of QoL predictors of patient satisfaction

**QLQ-C30 Scale**	**% of patients "very satisfied"**		**P - Value**
		
	**Below Median**	**Above Median**	
Global	88.8	94.6	0.03
Physical	88.3	95.0	0.01
Role	88.0	95.9	0.002
Emotional	91.7	92.3	0.79
Cognitive	90.0	94.7	0.07
Social	88.0	95.9	0.002
Fatigue	96.3	85.3	0.001
Nausea/Vomiting	94.7	87.0	0.005
Pain	94.6	89.3	0.04
Dyspnea	92.5	91.3	0.64
Insomnia	92.6	91.7	0.73
Appetite Loss	95.5	87.4	0.002
Constipation	92.9	90.4	0.37
Diarrhea	93.0	89.3	0.21
Financial Difficulty	94.9	89.1	0.01

### Multivariate Analysis - Predictors of Patient Satisfaction

In the first multivariate logistic regression model with only the QoL scores, only the fatigue score was a significant predictor of patient satisfaction (Table 8). Patients reporting more fatigue had significantly lower satisfaction. A patient reporting fatigue above the median (high fatigue) had reduced odds of 0.28 of being "very satisfied" compared to a patient with fatigue below the median (low fatigue). Overall, the model was significant (chi-square = 23.05, 9 df, p = 0.006).

**Table 8:** Multivariate logistic regression of predictors of patient satisfaction without controlling for demographic and clinical variables

**Variables**	**Odds Ratio**	**95% Confidence Interval**	**P - Value**
Physical	0.88	0.32 to 2.44	0.81

Role	1.17	0.36 to 3.82	0.80

Social	1.49	0.54 to 4.12	0.44

Fatigue	0.29	0.10 to 0.88	0.03

Nausea/vomiting	0.71	0.31 to 1.65	0.43

Pain	1.40	0.54 to 3.66	0.49

Appetite loss	0.60	0.25 to 1.44	0.25

Financial difficulty	0.61	0.28 to 1.34	0.22

Global health	0.71	0.28 to 1.85	0.49

Constant	326.15		0.01

In the second multivariate logistic regression model none of the demographic controls were significant (Table 9). Fatigue remained significant with an odds ratio of 0.28, so its effect was essentially unchanged when in a full model with controls. Again, the model overall was also significant (chi-square = 34.25, 19 df, p = 0.017). In Tables 8 and 9, "below median" is the reference category for all QoL scales.

**Table 9:** Multivariate logistic regression of predictors of patient satisfaction after controlling for demographic and clinical variables

**Variables**	**Odds Ratio**	**95% Confidence Interval**	**P - Value**
Age	1.04	0.99 to 1.08	0.16

Gender	0.83	0.28 to 2.53	0.78

Prior treatment history	0.81	0.34 to 1.92	0.62

Tumor type			0.24

Stage at diagnosis	0.59	0.23 to 1.50	0.26

Physical	1.56	0.47 to 5.14	0.47

Role	0.93	0.24 to 3.57	0.92

Social	1.18	0.38 to 3.65	0.77

Fatigue	0.28	0.09 to 0.91	0.03

Nausea/vomiting	0.67	0.26 to 1.72	0.40

Pain	1.61	0.56 to 4.57	0.38

Appetite loss	0.78	0.29 to 2.08	0.62

Financial difficulty	0.58	0.23 to 1.44	0.24

Global health	0.62	0.21 to 1.82	0.38

Constant	141.72		0.13

## Discussion

Patient satisfaction measures aim to assess the extent to which an individual's health care experiences match his or her expectations. Patients' perceptions of their care are an essential indicator of quality in health care and provide important clinical information about the extent to which a patient's needs and expectations are being met. We conducted this study to assess patient satisfaction with care at our cancer treatment hospital and determine its association with self-reported QoL.

Numerous studies on patient satisfaction with care have been conducted in an oncology setting. A study done in 91 patients with gastric and esophageal using the European Organization for Research and Treatment of Cancer 'satisfaction with in-hospital care' questionnaire (QLQ-SAT32) found the highest scores reported for doctors, nurses and overall satisfaction scales. The lowest scores were reported for access to the hospital, and comfort and cleanliness[8]. A study conducted in a prospective cohort of 39 patients with recurrent gynecologic malignancies receiving chemotherapy found that patient evaluation of care is more closely related to the interpersonal aspects of the health care provider relationship than it is to physical symptoms [13]. A national survey conducted in cancer patients in England for four common cancers: breast, colorectal, lung and prostate (55,674 patients) found that dissatisfaction was greater (p < 0.001) in younger and female patients and breast cancer patients expressed least, and prostate cancer patients expressed greatest dissatisfaction[11].

Yet another study conducted using Patient Satisfaction and Quality in Oncological Care (PASQOC)^® ^questionnaire in a random sample of 3384 cancer patients found that overall satisfaction was high, but specific reporting questions revealed many areas for improvement such as shared decision making, doctor-patient communication and organization of care. Patient-provider relationship, facility setting and information on diagnosis and treatment options were major determinants of a patient's willingness to recommend a facility to a friend or relative if needed [42]. Some studies found information received, technical competences, interpersonal and communication skills, time spent talking with doctors and nurses, accessibility and coordination of care, waiting times, and patients' emotional needs as important or priority areas to improve cancer care services [7,43,44]. In yet another study 'skills of nursing staff ', 'courtesy of nursing staff ', 'courtesy of people who drew blood' and 'cleanliness of hospital in general' were sought predictors of patients' overall perceptions of the quality of care [45].

We also evaluated the different determinants of patient satisfaction. In this study, we found that patient fatigue, as reported on the QoL fatigue scale, was a significant independent predictor of patient satisfaction. This finding has important implications for the care of new cancer patients given that fatigue is the most frequently reported symptom in cancer patients [46-51] with an estimated 60-96% of cancer patients undergoing treatment experiencing fatigue, including 60-93% of patients on radiotherapy and 80-96% of patients on chemotherapy [50,52]. There are several plausible mechanisms whereby increased fatigue could lead to lowered satisfaction. Increased fatigue may lead to a lowered capacity for tolerating normal issues that arise during care, such as the burden of paperwork during registration, time waiting for appointments, or procedures that are somewhat lengthy. In addition, increased fatigue can lead to lack of concentration and attention when interacting with medical personnel. All of these factors can, in turn, lead to a lower level of patient satisfaction.

Several studies in the literature have demonstrated the adverse impacts of fatigue on physical, emotional, economic and social aspects of cancer patients' lives [53-59]. A study conducted previously by our research group in 954 adult cancer patients found that after controlling for the effects of age and prior treatment history, deterioration in fatigue was statistically significantly associated with declines in health and physical, social and economic, psychological and spiritual, family, and global QoL[57]. Similarly, in a study of 1957 breast cancer survivors, fatigue was found to be significantly associated with high levels of depression, pain and sleep disturbance[55]. In another study conducted in 54 patients receiving adjuvant chemotherapy for breast cancer, fatigue showed strong significant correlations with emotional upset, muscle weakness, pain, numbness, sleep problems, problems with concentration and heartburn before the start of chemotherapy[58]. In a study conducted in a group of cancer patients undergoing radiotherapy, fatigue was associated with poor QoL. This association was considerably lower before treatment than at post-treatment or follow-up assessment, suggesting that fatigue becomes most important when treatment has ended[59]. The principal finding of our current study that fatigue independently predicts patient satisfaction takes the research on fatigue in cancer to the next level. Fatigue which is known to have an impact of QoL can also predict patient satisfaction, thereby, making it an important health care measure to evaluate and address.

In order to put our study in a better context of the existing literature, we also briefly review here several studies which have investigated the determinants of patient satisfaction in a variety of cancer care settings. A study conducted in 647 cancer patients using the EORTC IN-PATSAT32 questionnaire found that patients with a higher level than compulsory education or with a lower than a university education level reported lower overall satisfaction; patients reporting lower overall satisfaction were treated in a medical ward and had major compared to minor treatment toxicity. Patients with a relatively higher level of global health status reported higher level of satisfaction with doctors' and nurses' interpersonal skills, information provision and availability. In terms of satisfaction with care overall, patients treated in non-academic settings reported higher overall satisfaction compared to patients treated in academic/teaching settings [23]. Similarly, another study done in 2021 cancer patients at a specialized cancer hospital in Norway found that performance of nurses and physicians, level of information perceived, outcome of health status, reception at the hospital, older age and anxiety independently predicted 'patient satisfaction'[60]. Yet another study conducted in 2790 patients found that males, older and healthier patients tend to rate the overall quality of care higher than female, younger, and less healthy patients. Patients with prostate cancer tend to rate the overall quality of care higher than those with other cancer types [25]. Another study done in 2247 cancer patients in Ontario found that patients hospitalized once during the past 2 years reported significantly higher quality of care than those hospitalized three and four times (P < 0.05). Also, less healthy cancer patients (self-assessed health) tend to judge the quality of care lower than healthier cancer patients[45].

The results of our study do not compare directly with above mentioned studies because of the differences in study design, patient population studied, questionnaire used and outcome measures. Nevertheless, our study adds useful information to the growing body of literature on the importance of evaluating the predictors of patient satisfaction in oncology. Prospective studies evaluating the importance of QoL assessment and intervention on patient satisfaction should be carried out in a large population of cancer patients. Such studies will help us reach definitive conclusions on the role of QoL in improving patient satisfaction with overall cancer care. We are in the process of implementing a real-time computerized QoL collection and reporting system at our hospital, which will allow physicians to keep up to date with a patient's progress on QoL. A score below what is considered normal on any QoL domain would prompt specific action by the healthcare team.

Although this study reports on a relatively uncommon analysis of predicting patient satisfaction with QoL measures, several limitations of the study require acknowledgment. The patient cohort was limited to only those patients who were English speakers, so this study sample is therefore not broadly representative of cancer patients in general. The response rate for the study was relatively low compared to that reported in the literature [61]. This is because a significant fraction of the data was collected via a mailed survey. However, there was no difference in patient satisfaction by mode of data collection. The data we used for this study was not primarily meant for research purpose. CTCA is a unique medical center. It specializes in treating only cancer patients, and it has an intense focus on patient-centered care. Patients report very high levels of satisfaction with their care at CTCA. These results will not necessarily generalize to quality of life and its effects on cancer patient satisfaction with their care at general medical centers and hospitals. Patients in this study reported on their quality of life and satisfaction during or just after their first visit to CTCA. Thus, the results may or may not generalize to long-term patient satisfaction for patients undergoing care for cancer. Patient denial of dissatisfaction with health care may have influenced the results of this study [62]. Workers in the field of patient satisfaction have observed that patients may not express dissatisfaction with the provision of health care because of concern that the standard of care may deteriorate. In the event that such denial did occur in the study, the reported results overestimate the extent of patient satisfaction. This study used a non-validated patient satisfaction questionnaire. Finally, resilience might contribute to some of the findings observed in our study.

Our study has several strengths. We surveyed a large sample size of different cancer types. In determining patient satisfaction, the emphasis of care is generally on curing the patient and treating the disease rather than addressing the QoL of the patient and acknowledging the personal and social needs. It is well-documented that patient's psychosocial, social and financial needs [63-66], should also be taken into consideration and health care providers should be trained in adequate skills required to satisfy such needs.

## Conclusion

In summary, our study has demonstrated the predictive significance of fatigue in evaluating patient satisfaction in oncology. To the best of our knowledge, this study is the first in the literature to report on the role of QoL as a predictor of patient satisfaction in a large heterogeneous sample of cancer patients.

## Competing interests

The authors declare that they have no competing interests.

## Authors' contributions

CGL was the main author of the manuscript, participated in concept, design, data analysis and data interpretation. MR and JFG participated in statistical analysis, data interpretation and writing. DG participated in concept, design, statistical analysis, data interpretation and writing. All authors read and approved the final manuscript.

## Pre-publication history

The pre-publication history for this paper can be accessed here:


